# CD-tagging-MS2: detecting allelic expression of endogenous mRNAs and their protein products in single cells

**DOI:** 10.1093/biomethods/bpx004

**Published:** 2017-05-12

**Authors:** Jonathan Sheinberger, Hodaya Hochberg, Erez Lavi, Itamar Kanter, Shira Avivi, Gita Reinitz, Avital Schwed, Yuval Aizler, Eli Varon, Noa Kinor, Yaron Shav-Tal

**Affiliations:** The Mina & Everard Goodman Faculty of Life Sciences & Institute of Nanotechnology, Bar-Ilan University, Ramat Gan, 5290002, Israel

**Keywords:** gene expression, transcription, mRNA detection, stress granules, allelic expression

## Abstract

Discriminating between the mRNA and protein outputs of each of the alleles of an endogenous gene in intact cells, is a difficult task. To examine endogenous transcripts originating from a specific allele, we applied Central Dogma tagging (CD-tagging), which is based on a tag insertion into an endogenous gene by creation of a new exon. Previously, CD-tagging was used to tag endogenous proteins. Here we developed a CD-tagging-MS2 approach in which two tags were inserted in tandem; a fluorescent protein tag in conjunction with the mRNA MS2 tag used for tagging mRNAs in cells. A cell clone library of CD-tagged-MS2 genes was generated, and protein and mRNA distributions were examined and characterized in single cells. Taking advantage of having one allele tagged, we demonstrate how the transcriptional activity of all alleles, tagged and untagged, can be identified using single molecule RNA fluorescence *in situ* hybridization (smFISH). Allele-specific mRNA expression and localization were quantified under normal and stress conditions. The latter generate cytoplasmic stress granules (SGs) that can store mRNAs, and the distribution of the mRNAs within and outside of the SGs was measured. Altogether, CD-tagging-MS2 is a robust and inexpensive approach for direct simultaneous detection of an endogenous mRNA and its translated protein product in the same cell.

## Introduction

RNA detection and quantification in intact cells has come of age, from the days of crude RNA detection by methyl-green pyronin [1], through the identification of specific RNAs by electron microscopy [2–5] and *in situ* hybridization [6]. The development of fluorescence *in situ* hybridization (FISH) [7] has since offered a powerful quantitative and qualitative tool for gene expression studies on the single cell [8] and single molecule levels [9], providing spatial, and in time-course experiments, temporal information on RNA localization in cells [10].

RNA FISH has been modified to produce a variety of mRNA detection approaches that vary in sensitivity, specificity, and resolution. An important step in FISH probe development was made by Singer and colleagues who increased probe specificity and signal, allowing the detection of mRNA at the single molecule level, in single cells [9]. Various approaches are now available for single molecule detection of RNA by FISH (smFISH) [11–16], enabling the quantification of mRNAs and providing a picture of the transcriptional profile of a certain gene of interest within single cells and tissues [17]. For instance, smFISH allowed the quantification of different alternative spliced mature mRNAs [18], and applying smFISH to study introns, showed that alternative splicing can occur post-transcriptionally [19], as opposed to co-transcriptional splicing [20]. Development of color-based multiplexing to visualize several mRNA species simultaneously in smFISH [8, 21–23] has made significant progress, providing even the visualization of up to 1000 different mRNAs in a single cell [24]. Multiplex detection of mRNAs showed that mRNAs travel as separate entities [25].

Detection of specific mRNA species within intact cells could also be obtained using an RNA binding protein (RBP) approach, referred to now as MS2 mRNA-tagging [26]. In this approach, the mRNA is usually expressed from an exogenous gene construct, using short DNA sequences (MS2 repeats) that are inserted in tandem into the gene of interest. Upon transcription, the MS2 repeats form stem-and-loop structures in the mRNA that are recognized and bound by an RBP, the MS2 binding protein (or MS2 coat protein, MCP) [27, 28]. Detection of MS2-tagged mRNAs requires the insertion of multiple MS2 repeats into the 3′ UTR of the studied gene, and this has usually been performed using exogenous plasmids. At first, large tandem genomic insertions were generated in cells in order to detect the actively transcribing genes [29], but later the detection of single genes could be monitored [30–32]. In fact, the generation of an MS2 knock-in mouse proved the robustness of the MS2 system for the detection of endogenous mRNA in mammalian cells [31, 33–35]. The MS2 tag is also useful for detection of mRNA molecules in RNA FISH experiments since a probe hybridizing with this repetitive region will bind many times, and enhance the signal detected [30].

In order to generate an affordable, high-throughput, endogenous mRNA tagging system for mammalian cells, which will simultaneously also allow the detection of the protein product, we harnessed the central dogma tagging (CD-tagging) technique [36] to perform MS2 and yellow fluorescent protein (YFP) genomic insertions. CD-tagging utilizes engineered retroviral constructs for the stable integration of exogenous DNA into the mammalian genome, and was used to insert YFP sequences into the genome, achieving endogenous protein labeling [37, 38]. This approach is different than gene trapping which generates a truncated mRNA and protein from the tagged gene [39]. In order to simultaneously track the endogenous protein together with the earlier step of gene expression, namely mRNA transcription, we used a CD-tag that combined a YFP coding sequence to tag the endogenous protein, as well as the MS2 repeats (24×MS2 repeats as a coding sequence) to generate the mRNA tag. The YFP-24×MS2 sequence was flanked with consensus splice sites that would mark it as a new exon, and since the retroviral genome usually integrates into intronic regions of active genes, the new exon was anticipated to become part of a mature mRNA. We termed this approach CD-tagging-MS2. We expected the integrated MS2 sequences to provide robust detection of mRNA by RNA FISH using a generic probe to the MS2 region instead of generating expensive and unique probe sets for each endogenous gene studied.

The fact that in CD-tagging-MS2 only one of the alleles of a certain gene is tagged, allows the comparative detection of different alleles and their mRNA products in the same cell, for the examination of allelic expression. Previously, allelic expression has been difficult to study due to shortage in allele-discriminating methods. Currently, single nucleotide polymorphism (SNP)-arrays [40–42], RNA-seq [43, 44] and a few versions of RNA FISH protocols [45–47] that utilize specialized probes designed for each studied gene, allow the quantification of transcripts with respect to their allelic origin in cells and tissues. However, visualizing and discriminating between the alleles and the transcribed mRNAs using RNA FISH approaches that rely on detection through the combinatorial use of many probes is quite challenging. In these cases, the signal levels are very low since only one or few probes can differentiate between allelic transcription on the single nucleotide level. In general, these studies have indicated that many autosomal genes are not bi-allelic in expression as previously thought, and are rather considered dynamic autosomal random mono-allelic expressed (aRME) genes [48]. The randomness is attributed to independent stochastic transcription from each allele. The CD-tagging-MS2 approach we present is capable of generating a clear cellular picture of allelic mRNA expression using standard probe sets.

## Materials and methods

### Plasmids

To create the library constructs, a pBabeAE-YFP plasmid (provided by Uri Alon, The Weizmann Institute of Science) was used as a backbone to which we added YFP-24×MS2 (two versions of MS2) and Cerulean-24×MS2 (version one), following the removal of the original YFP sequence using EcoRI and BamHI restriction enzymes. The helper plasmid (pSV-ψ-E-MLV), used in synergy with the pBabe plasmid for the purpose of virus packaging, was a gift from Doron Ginsberg [Bar-Ilan University (BIU)].

### Modifying the MS2 sequence repeats for CD tagging

Two types of YFP-24×MS2 sequences were generated. First YFP-24×MS2: 5′-GATCCTAAGGCACC TAATTGCCTAGA AAACATGAG G A T CACCCATGTCTGCAGGTCGAC TCTAGAAAACATGAGG ATCA C CCATGTCTGCAGTATTCCCGGGTTCATT-3′. Bioinformatics analysis showed that removal of the adenine nucleotide in the 109th position of the MS2 sequence generated a reading frame without stop codons. This modification did not affect the loop segments, and was the smallest modification possible yielding a completely translatable MS2 sequence. To examine whether the MS2 sequence contained potential splicing sites, we used the Human Splicing Finder program [49]. A strong 5′ splice site (Splice Donor) was identified at the beginning of the MS2 sequence. We then changed one nucleotide located at position 11 of an MS2 single repeat to significantly reduce the probability of splicing. As a final step before construct synthesis, we used the online splicing prediction software Genscan [50] that predicts the mature mRNA sequence by use of splicing scoring algorithms. As a test sequence, we entered the viral MS2 insert into intron 1 of the *actin cytoplasmic 1* (*ACTB*) gene, to create one continuous sequence as expected to occur during CD-tagging-MS2, and then applied Genscan. Initially, the program showed that the YFP-24×MS2 sequence was correctly spliced, however, with low probability. Therefore, we modified the splice sites until reaching a sequence with high probability of splicing. This sequence was also used to create the Cerulean-24×MS2 construct.

Second YFP-24×MS2: 5′-GATCCTAAGGTACCTAA TTGCCT AG AAAGCACGAGCATCAGCCGTGCCTCCAGGTCGA ATCT TCAA ACG ACGACGATC ACGCGTCGGTCTGCAGT ATTCCCGGGTTCATTA-3′. The second sequence was based on a stable MS2 sequence that underwent sequence optimization to eliminate repeat disposal (Addgene plasmid No. 31865). We kept the original stem-loop regions and changed only the sequences that were in between or flanking the stem-loop structures, creating a hybrid MS2 sequence.

The YFP-24 MS2 first and second sequences versions were synthesized (by Genscript) and ligated into pBabeAE-YFP following the removal of the original YFP coding sequence using BamHI and EcoRI. A BglII restriction site was placed between the YFP and the MS2 repeats for future changes of the MS2 or/and the fluorophore. To create clones that were tagged with Cerulean instead of YFP, the pBabeAE-YFP-24 MS2 first version was cut with EcoRI/BglII to remove the YFP coding sequence, and the adjacent 3′ splice site (SS) was replaced with a 3′ SS-Cerulean sequence that was produced via polymerase chain reaction (PCR) using the following primers: reverse primer 5′-ATA AGA TCT CTT GTA CAG CTC GTC CAT-3' and forward primer 5′-ATA GAA TTC TAA CTA ATC TCC TCT CTT CTC CTC TCT CCA GGT GAG CAA GGG CG- 3'.

### Cell culture

Human U2OS ecotropic cells (for generation of the library clones), were maintained in low glucose Dulbecco’s modified Eagle’s medium (DMEM, Biological Industries, Israel) containing 10% fetal bovine serum (FBS, HyClone). HEK 293T cells were maintained in high glucose DMEM (Gibco) supplemented with 10% FBS. The transgenic retroviruses that were used to produce the library clones were generated in HEK 293T cells. To form stress granules (SGs), arsenite (1 mM) was added to the medium for up to 45 min. For recovery purposes, the medium was aspirated and replaced with fresh medium for 3 h.

### Retrovirus packaging and infection

Day 1: 18–24 h prior to transfection, HEK 293T cells were plated in a 10 cm dish at 40% confluence. Day 2: Cells were co-transfected with the pBabeAE-YFP-24 MS2 and helper plasmids using calcium phosphate transfection and were then incubated at 37 °C (5% CO_2_) for 8 h. Then the medium was replaced with 10 mL fresh medium and cells were incubated overnight. Day 3: To collect the virus supernatant, the medium was replaced with 5 mL of fresh medium and collected into a 4 °C cooled tube 10 h later. This was repeated three more times during the next 36 h. Day 4: 16 h prior to infection, U2OS ecotropic cells were plated in a 10 cm dish at 20% confluence. Day 5: The virus supernatant was centrifuged at 1 rpm for 5 min to pellet the cell debris, and the supernatant was collected. Then, 3 mL of virus supernatant was added to the U2OS ecotropic cells followed by 1.5 mL of low glucose DMEM medium supplemented with 4.5 µL (8 mg/mL) of hexadimethrine bromide [Polybrene (Sigma)]. Then, 3–6 h later, 5.5 mL of fresh medium was added and the cells were incubated overnight. Following incubation, the medium was replaced with 10 mL of fresh medium, and the cells were incubated for 72 h for the expression of the fluorescent protein (YFP) and subsequent fluorescence activated cell sorting (FACS).

### FACS

The YFP positive library clones were sorted using a FACSVantageSE sorter (BD Biosciences). 10^6^ U2OS ecotropic-infected cells were levitated in 1 mL of low glucose DMEM supplemented with 20% FBS, streptomycin and HEPES buffer. The Cerulean positive clone was sorted using a FACSAriaIII sorter under the same conditions.

### RNA extraction and rapid amplification of cDNA ends

Total RNA was produced using Tri-Reagent (Sigma) and DNA was removed using Turbo-DNase free kit (Ambion). 3' rapid amplification of cDNA ends (RACE) was performed using RevertAid™ First Strand cDNA Synthesis Kit (Fermentas) using an oligo-dT-adapter hybrid primer Qt. The cDNA was used as a template for nested PCR using Promega’s Go-Taq green master mix (M712B). To perform the nested PCR, adapter-anti sense primers (Q-out and Q-in) and a variety of YFP sense primers (gene-specific primer) were used. The amplified segments were excised from the agarose gel for sequencing. The primers sequences are (in accordance with Fig. 2a):

Qt: 5′-CCA GTG AGC AGA GTG ACG AGG ACT CGA GCT CAA GCT TTT TTT TTT TTT TTT T-3' (not illustrated in Fig. 2a)

Qo: 5′-CCA GTG AGC AGA GTG ACG-3' (illustrated as ‘3p’ in Fig. 2a)

Qi: 5′-GAG GAC TCG AGC TCA AGC-3' (illustrated as ‘3p’ in Fig. 2a)

Primer -3: 5′-CAA GGA CGA CGG CAA CTA CAA GAC C-3'

Primer -4: 5′-CGA CAA GCA GAA GAA CGG CAT CAA G-3'

Primer -5: 5′-GGA TCA CTC TCG GCA TGG AC-3'

Some gel purifications required further handling prior to sequencing. The excised bands were cloned into the pGEM T-easy vector kit (Promega) before sequencing. For 5′ RACE, the ExactSTART™ Eukaryotic mRNA 5′ & 3′-RACE Kit was used (Epicentre) to identify the IPO7-YFP-20 MS2 clone, and the 5′/3′ RACE Kit 2nd Generation (Roche) was used to identify the ANLN-YFP-12 MS2 clone. The sense primer in the case of IPO7 was: 5′-TCA TAC ACA TAC GAT TTA GGT GAC ACT ATA GAG CGG CCG CCT GCA GGA AA-3′. The sense primer in the case of ANLN was: 5′-GAC CAC GCG TAT CGA TGT CGA C-3′

The anti-sense primers sequences in both cases were:

Primer -1: 5′-TTC AGG GTC AGC TTG CCG TAG G-3'

Primer -2: 5′ GTC TTG TAG TTG CCG TCG TCC TTG-3′

### DNA-based assay for assessing MS2 repeat numbers

For some of the clones, extracted RNA containing genomic DNA was used as a DNA template in PCR. 1 µL of nucleic acid was used as the DNA template in PCR. For most of the clones, DNA extraction was performed using the ArchivePure DNA Cell/Tissue and Tissue Kits (5 PRIME) as a template. Each clone’s DNA was diluted 10-fold and 1 µL was used for PCR. The primers that flanked the YFP-xMS2 repeats segment were: sense primer 5′-CGC GTC ACC TTA ATA TGG-3′ and anti-sense primer 5′-CTT GAA CCT CCT CGT TCG-3′.

### Splicing assay

RevertAid™ First Strand cDNA Synthesis Kit (Fermentas) was used to prepare the cDNA from RNA extracted from the tagged clones: IPO7, LIM and SH3 Protein 1 (LASP-1) and hnRNP A1. Primers used:

IPO7: exon 1 (sense)- 5′- ATT CCT GGC CCA GTA GCA-3′, YFP (anti-sense)- 5′ GTC TTG TAG TTG CCG TCG TCC TTG-3′, YFP (sense)- 5′-CGA CAA GCA GAA GAA CGG CAT CAA G-3′, exon 2 (anti-sense)- 5′-CAC AGG TAA ATC CAG CTG-3′, exon 3 (anti-sense)- 5′-GGA GAA TGG ATA ATG GCT TC-3′, exon 4 (anti-sense)- 5′-GCC AAC AAG CAC TGT TAT-3′, exon 6 (anti-sense)- 5′-TCC CTG TTC ACA ACA GTC-3′, exon 7 (anti-sense)- 5′-TTC CAC CAT GGT AAC TCA-3′, exon 8 (anti-sense)- 5′-TGG ACA CCA ACA GCA ATG C-3′, exon 9 (anti-sense)- 5′-CTT CCA GGT GAG AGC ATG AG-3′, exon 10 (anti-sense)- 5′-TGC CAA AGT TCC TCA TCA-3′.

LASP-1: exon 1 (sense)- 5′-GGT GCG GCA AGA TCG TGT-3′, YFP (anti-sense) – 5′ GTC TTG TAG TTG CCG TCG TCC TTG-3′, YFP (sense)- 5′-CGA CAA GCA GAA GAA CGG CAT CAA G-3′, exon 2 (anti-sense)- 5′-CAG TGT CAT CTT GCA GGT-3′, exon 3 (anti-sense)- 5′-GAC TCT GGA GCT CAC TCT-3′, exon 4 (anti-sense)- 5′-TCG GGC GTG TCT GCC ACT-3′, exon 5 (anti-sense)- 5′-GGT GAG GCT GCT GCT GCT-3′, exon 6 (anti-sense)- 5′-GGC GCT GCG CTG TAT GGA-3′, exon 7 (anti-sense)- 5′-CTC CAC GTA GTT GGC CGG-3′.

hnRNP A1: exon 2 (anti-sense)- 5′-GTC CGT GAG CGT TCC CCA-3′, exon 5 (anti-sense)- 5′-TGG CTG GAT GAA GCA CTA-3′, exon 9 (anti-sense)- 5′-CTT GGT TTC GTG GTT TTG-3′, exon 11 (anti-sense)- 5′-CTA CAC CAA GGT TTC CGA-3′, YFP (sense)- 5′-CGA CAA GCA GAA GAA CGG CAT CAA G-3′.

### Real-time qPCR

Total RNA was extracted from cells using the AurumTM Total RNA mini kit (Bio-Rad Laboratories Inc). After reverse transcription using qScript cDNA Synthesis Kit (Quanta Biosciences), cDNA was amplified using the following primer pairs:

IPO7: Forward primer- GATGGACCCCAACACCATTA, Reverse primer- ATGTCGGAACAGCTGGATTT.

MS2: Forward primer- TCCCGGGTTCATTGCAAAG, Reverse primer- TGCCTGTCTCACAGGTAAA.

Tubulin: Forward primer- GCCTGGACCACAAGTTTGAC, Reverse primer- TGAAATTCTGGGAGCATGAC.

Glyceraldehyde 3-phosphate dehydrogenase (GAPDH): Forward primer- TCT TCC AGG AGC GAG ATC CCT, Reverse primer- TGC AAA TGA GCC CCA GCC TTC T.

Real-time qPCR was performed using the primers listed above, and PerfeCTa® SYBR® Green FastMix®, ROX™ (Quanta Biosciences) according to the manufacturer’s protocol on a CFX-96 system (Bio-Rad). Analysis was performed with Bio-Rad CFX manager software. Relative levels of RNA expression were measured as the ratio of comparative threshold cycle (CT) to internal control (GAPDH and tubulin) mRNA.

### Western blotting

Cells were washed in cold Phosphate-buffered saline (PBS) and proteins were extracted in immunoprecipitation (IP) lysis buffer (Pierce) containing 10 mM Na-flouride, 1 mM Na-orthovanadate, protease inhibitor cocktail (Sigma) and 1 mM phenylmethylsulfonyl fluoride (PMSF), and placed on ice for 15 min. The resulting lysate was centrifuged at 10 000 rpm for 10 min at 4 °C. 30 μg of protein was run on SDS-polyacrylamide gels and transferred to a nitrocellulose membrane (0.45 μm). The membrane was blocked in 5% skim milk (BD) prior to anti-GFP (Santa Cruz), and with 5% BSA prior to rabbit anti-ANP32A (Abcam), rabbit anti-tubulin (Abcam) and rabbit anti-LASP-1 (Abcam) primary antibodies for 2 h at room temperature (RT), followed by incubation with an HRP-conjugated goat anti-rabbit IgG (Millipore) for 1 h at RT. Immunoreactive bands were detected by the enhanced chemiluminescence kit (ECL, Pierce).

### Protein localization tests

The hnRNPA1 CD-tagged clone was tested for hnRNP A1 localization into cytoplasmic SGs under oxidative stress induced by arsenite (1 mM, 45 min), as previously published [51]. The nucleolin CD-tagged clone was tested for nucleolin re-localization from nucleoli into the nucleoplasm following transcriptional inhibition by actinomycin D (ActD) (5 µg/mL, 140 min) [52]. The LASP-1 CD-tagged clone was tested for nuclear export inhibition in response to the export inhibitor Leptomycin B (10 ng/mL, 6 h).

### Fluorescence *in situ* hybridization

LASP-1-YFP-10×MS2, ANLN-YFP-12×MS2 and IPO7-YFP-20×MS2 tagged clones were grown on coverslips and fixed for 20 min in 4% paraformaldehyde (PFA), and left overnight in 70% ethanol at 4 °C. The next day, the cells were washed with 1× PBS and then incubated for 10 min in 40% formamide (4% saline-sodium citrate (SSC). Fluorescently-labeled (Cy3) DNA probes that target the MS2 sequence (∼10 ng probe, 50-mer) were hybridized overnight at 37 °C in a dark chamber in 40% formamide. The next day, cells were washed twice with 40% formamide for 15 min and then washed for 2 h with 1× PBS. Nuclei were counterstained with Hoechst 33342 and coverslips were mounted in mounting medium. Probe sequence was (50-mer, one probe per MS2 cassette labeled with five fluorophores):

5′-CTAGGCAATTA GGTACCTTAGGATCTAATG AACCCGGGAATAC T G CAGAC-3′.

For poly(A) RNA detection in the hnRNP A1-YFP clone under oxidative stress, fluorescently-labeled (Cy5) DNA poly-dT probes that target the poly(A) sequence (∼10 ng probe, 50-mer) were used in 15% formamide.

FISH experiments with Stellaris (Biosearch Technologies) probes were performed according to the manufacturer’s adherent cell protocol. Probes used were: Cy3-labeled MS2 (20-mer probes labeled with a single fluorophore, three probes per MS2 cassette), Cy5-labeled YFP and Cy5-labeled IPO7. To perform the allele-specific detection, two probes were mixed in the same hybridization buffer tube. To reduce photobleaching, the cells were submerged in glucose oxidase (GLOX) buffer (pH = 8, 10 mM, 2× SSC, 0.4% glucose), supplemented with 3.7 ng of glucose oxidase (Sigma G2133-10KU) and 1µl Catalase (Sigma 3515) prior to imaging [11, 53].

### Quantitative RNA FISH

The smFISH protocol was adopted from [54] with minor modifications. Following FISH experiments, 3D stacks of cells were taken using a wide-field fluorescence microscope at 100× magnification. Specifically, 53 Z planes were acquired for each cell with 250 nm steps. After acquisition, the images underwent deconvolution using Huygens software and were transferred to Imaris software for image processing. In Imaris, the signal of each mRNA spot was evaluated using “surface objects”. Then, the common value for the fluorescent signal was defined as the amount of signal of a single mRNA molecule. To evaluate the number of nascent mRNAs on the transcription site, the fluorescent signal measured on the site was divided by the value of a single mRNA. To detect mRNA originating from the tagged allele, spots from both the Cy5 and Cy3 channels were co-localized using the “spots co-localize” option in Imaris. The threshold value used was 0.4, which was determined after screening hundreds of co-localization events. Overall, 40 IPO7-tagged cells and 38 U2OS control cells that were collected in four separate experiments were used for quantification.

### Immunofluorescence and SG quantification

Following RNA FISH, the cells were treated with 0.5% Triton X-100 for 2 min followed by 3× PBS washes. The cells were then stained with the indicated antibodies for 1 h, washed 3× in PBS and then incubated with the appropriate secondary antibodies for 1 h. Primary antibody used was mouse anti-G3PB1 (Abcam) and the secondary antibody was goat anti-mouse 488 (Abcam). To quantify SG numbers, 80 cells were analyzed. The analysis was performed using ImageJ according to a previous study [55]. Overall, for arsenite treatment analysis, 8391 IPO7-mRNAs and 889 SGs molecules, were examined.

### Fluorescence microscopy and live-cell imaging

Wide-field fluorescence images were obtained using the Cell^R system based on an Olympus IX81 fully motorized inverted microscope (60X PlanApo objective, 1.42 NA) fitted with an Orca-AG CCD camera (Hamamatsu) driven by the Cell^R software. Live-cell imaging was carried out using the Cell^R system with rapid wavelength switching. For time-lapse imaging, cells were plated on glass-bottomed tissue culture plates (MatTek, Ashland, MA, USA) in medium containing 10% FBS at 37 °C. The microscope is equipped with an incubator that includes temperature and CO_2_ control (Life Imaging Services, Reinach, Switzerland). For long-term imaging, several cell positions were chosen and recorded by a motorized stage (Scan IM, Märzhäuser, Wetzlar-Steindorf, Germany). Mitosis movies were recorded overnight by capturing images at selected areas every 30 min. Some of the mitosis movies were acquired using an Olympus IX81 microscope (636 Plan-Apo, 1.4 NA) equipped with an EM-CCD (Quant-EM, Roper) and an XY&Z stages (Prior), driven by MetaMorph (Molecular Devices). Experiments were performed at 37 °C with 5% CO_2_ using a live-cell chamber system (Tokai).

### shRNA treatment

The shRNA plasmids (p53 and GFP) and the mCherry plasmid were a kind gift from Dan Canaani (Tel-Aviv University). The shRNA plasmids, p53 and GFP, were co-transfected with the mCherry/Cerulean plasmid using Lipofectamine 2000 (Invitrogen).

### Poisson distribution

Poisson distribution was used to model the number of random independently occurrences of a phenomenon in an interval of time or space. In our case, it was used to model the number of times that mRNA will be found in a given SG We used the maximum-likelihood estimator (MLE) to fit the number of mRNAs in SGs to the Poisson distribution. The fit was done using the *fitdistr* in the MASS package in R. This function used an analytical closed-form MLE for parameter estimation. Overall, 889 SGs and 1040 mRNAs were integrated in the analysis.

## Results

### A system for endogenous tagging of an mRNA and its protein in the same cell

**Figure 1:**
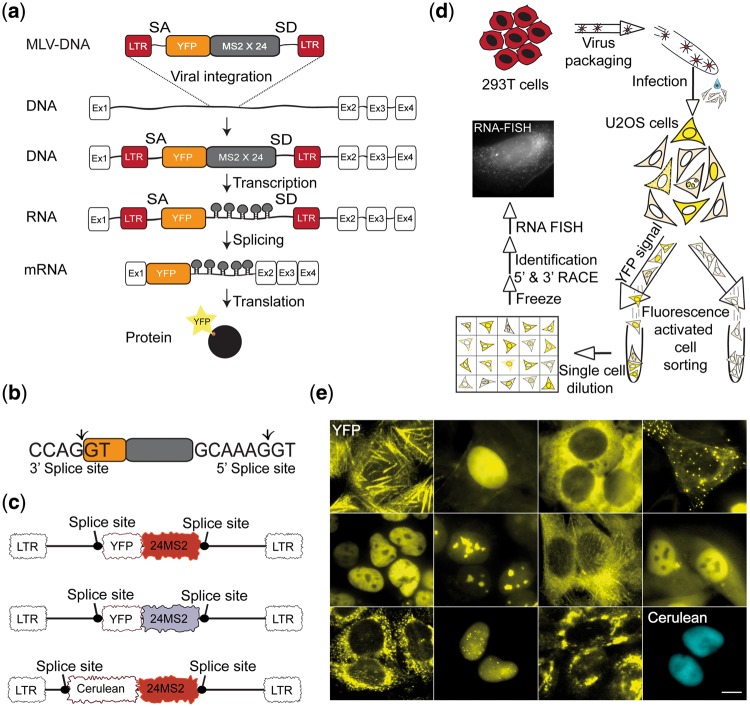
The CD-tagging-MS2 system**.** (**a**) Scheme of the CD-tagging-MS2 approach showing the viral construct containing the YFP tag followed in frame by the 24×MS2 repeated sequence, and how it integrates in an intron to finally transcribe as a new exon, to translate into a CD-tagged protein. SA—splice acceptor; SD—splice donor; MLV; YFP coding sequence; 24×MS2—24 repeats of the MS2 sequence; LTR-MLV long terminal repeats. (**b**) The selected 5′ and 3′ splice consensus sequences flanking the YFP-24×MS2 sequence. (**c**) An illustration of the DNA constructs that were used to generated CD-tagged-MS2 cells: first generation of MS2 repeats (red) containing the original MS2 sequence and the second generation MS2 optimized repeats sequence (gray) to avoid loss of repeats during bacterial transformation. A construct in which a Cerulean fluorescent protein exchanged the YFP, was also generated (cyan). (**d**) The CD-tagging-MS2 workflow; HEK 293T cells were used as host cells for the retroviruses. Then, infections on ecotropic U2OS cells were performed and cells which were positive for the YFP tagged protein were selected and purified using FACS. The YFP-positive clones were then diluted and seeded as single cells in a 96-well plate. The final phase included freezing, imaging, and gene identification using 3′ and 5′ RACE. (**e**) Representative images of some of the library clones tagged with YFP-24×MS2 or Cer-24×MS2. Scale bar = 10 µm.

CD-tagging uses engineered retroviral vectors that contain a gene sequence serving as a tag, which is flanked on either side by splice sites (SS). Following infection, the vector randomly integrates into the genome, and when the integration site falls within an intron, then a new exon is formed within an endogenous gene. CD-tagging studies have shown that most integration events occur near the N-terminus of the resultant tagged protein [38], probably due to the generally large size of the first intron [56, 57] and the preference of murine leukemia virus (MLV) to integrate near the beginning of genes [58]. For CD-tagging-MS2, we combined in-frame and downstream to the YFP coding sequence also a 24×MS2 repeated sequence that contained a coding region, to produce a new CD exon that would integrate into the coding region of endogenous proteins (Fig. 1a). The YFP-24×MS2 coding sequence contained SSs on each side (Fig. 1b) but could not form a protein independently since it lacked a start codon, therefore, a fluorescently tagged protein would only appear if in-frame splicing had taken place.

CD-tagging-MS2 also produces an mRNA tag. We prepared two versions of the MS2 sequence repeats for these integrations, since repeated sequences are known to be randomly removed during bacterial transformation procedures due to recombination events, and it was unclear how the repeats would behave during viral infection into the genome. The first version of the MS2 repeats is known for repeat discarding but has been frequently used in many studies, while the second type of repeats underwent sequence optimization to reduce excision but kept the original stem-loop regions that are bound by the MCP (Fig. 1c). In addition, the first MS2 repeats version was also integrated with a Cerulean fluorescent protein, to examine the ability to detect expression of different colors (Fig. 1c). These DNA gene cassettes were sub-cloned into the viral pBabe construct, and used to infect U2OS cells, until a library of CD-tagging-MS2 positive clones was obtained (Fig. 1d).

### Characterizing and identifying the CD-tagged-MS2 genes

**Figure 2:**
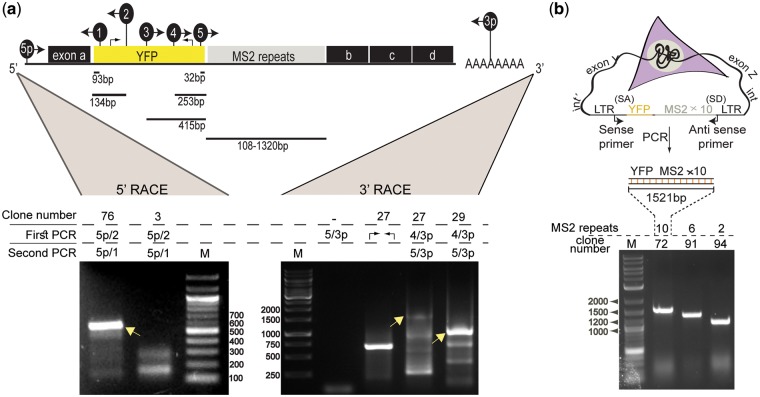
Library clones identification using 3′ and 5′ RACE and MS2 repeats assessment. (**a**) Scheme of a tagged mRNA (top) depicting the YFP-MS2 tag as a new exon between original exons a and b. The location of the primers used in both 5′ and 3′ RACE are shown and numbered. Example of the 5′ RACE reaction (bottom left) performed on clone IPO7-20×MS2 showing primers used in the first PCR reaction and in the nested PCR reaction, and the band that was excised and sent for sequencing (arrow). 3′ RACE (bottom right) shows the primers and bands (arrows) that revealed the identity of clone hnRNP A1-4×MS2 and clone nucleolin-4×MS2. (**b**) DNA-based PCR to assess MS2 repeat numbers on the genomic level. An illustration (top) of the PCR primers used for amplifying the MS2 containing region in the genome, and an example of an agarose gel (right) showing amplified regions obtained from three clones with different sizes of MS2 repeats, as marked above.

Initial detection of positive clones was performed by YFP or Cerulean observation of the endogenously tagged proteins (Fig. 1e). A battery of YFP-positive clones showed a wide variety of protein distributions, as expected. We also found that Cerulean is detected successfully. Gene identification was performed by 5′ and 3′ RACE followed by sequencing (Fig. 2a). Gene identities are listed in Supplementary Table S1 showing that most integrations occurred within the first intron of the tagged genes. MS2 repeat loss was observed in the sequences of the identified genes using both versions of the MS2 repeated sequences, with a tendency to obtain clones with lower numbers of MS2 repeats than 24. We estimated MS2 repeat numbers in unidentified clones prior to RACE using a genomic PCR assay (Fig. 2b). To verify that the 24×MS2 repeats were indeed packaged into the viruses without repeat loss, a HEK 293T cell line which stably expressed the CD-tagging 24×MS2 cassette, underwent RNA FISH and PCR, which confirmed that the whole cassette was encapsulated (Supplementary Fig. S1). Examination of the splicing pattern of several clones containing different numbers of MS2 repeats showed that the predicted splicing events were sustained (Supplementary Fig. S2).

Previous CD-tagging studies have shown that the YFP tag usually did not interfere with correct protein localization. We tested whether the YFP-24×MS2 coding region influenced protein localization. The ability of tagged cells to undergo productive cell division showed that mitosis events occurred with normal behavior and frequency (Supplementary Fig. S3 and Supplementary Video S1). Overall, all the identified clones showed localization patterns identical to their wild-type counterparts, examined in respect to published immunofluorescence (IF) data. Since we could work with living cells, we tested whether protein localization dynamics were preserved in several clones that bear unique localization patterns. Following are some examples.

**Figure 3:**
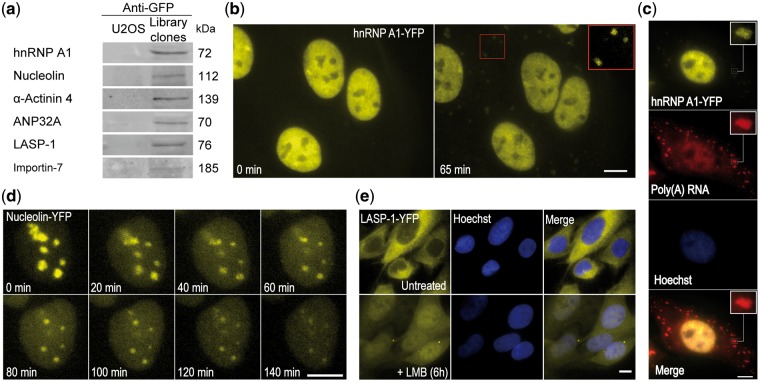
Characterizing the dynamic localization of tagged proteins. (**a**) Western blots using anti-GFP, showing the expected molecular weights of the tagged proteins. (**b**) The formation of SGs as observed using hnRNP A1-YFP-4×MS2 tagged cells during arsenite treatment (1 mM, 45 min). Red rectangle shows an enhanced image of SGs, using ImageJ for optimal visualization. (**c**) Confirming the identity of the cytoplasmic bodies as SGs by applying RNA FISH with Cy5-fluorescent oligo (dT) probes that label poly(A) RNA. Images inside the white rectangles were enhanced using ImageJ for optimal visualization. (**d**) Nucleolin-YFP-4×MS2 protein translocation to the nucleoplasm in response to ActD (5 μg/mL) treatment for 2 h. (**e**) LASP-1-YFP-10×MS2 shuttling properties were diminished after exposure to the protein export inhibitor agent LMB (1 mM, 6 h). Scale bar = 10 µm.

The hnRNP A1 protein tagged in the hnRNP A1-YFP-4×MS2 clone is an abundant mRNP component belonging to the heterogeneous ribonucleoprotein (hnRNP) family [59]. hnRNP A1 RBP is involved in splicing and in post-splicing activities such as mRNA export and cap-dependent internal ribosome entry site-mediated translation [51]. The hnRNP A1-YFP-4×MS2 tagged protein product was predicted to yield a ∼72 kDa band in western blotting (Fig. 3a), which represents the wild-type protein plus the YFP and the 4×MS2 repeats regions. Western blotting performed on other clones from the library with an anti-GFP antibody, validated the tagged allele’s ability to generate a full-sized protein product. Western blotting with antibodies that could recognize both the wild-type and tagged proteins, also showed that the tagged protein was translated (Supplementary Fig. S4). We note that translation efficiency needs to be tested for each case.

The localization of the tagged hnRNP A1 protein was predominantly nuclear, as the endogenous protein (Fig. 3b). SGs are cytoplasmic bodies consisting of proteins and mRNAs, which appear following exposure to environmental stress. SGs are most probably involved in mRNA storage, degradation, and translation re-initiation [60]. hnRNP A1 relocates to SGs under cellular stress [51], specifically, following oxidative, osmotic shock (OSM) and heat shock stresses. Localization dynamics of the tagged hnRNP A1 in living cells exposed to oxidative stress (induced with 1mM arsenite) [51] showed relocation into SGs after 45 min of treatment, as expected (Fig. 3b). To validate that the SGs contained mRNAs as expected, we performed RNA FISH using an oligo(dT) probe, which hybridizes to all poly(A) RNAs, and observed co-localization of tagged hnRNP A1 and poly(A) RNA under oxidative stress in SGs (Fig. 3c).

The nucleolin protein tagged in the nucleolin-YFP-4×MS2 clone is a component of the nucleolus, and is involved in ribosomal RNA processing. ActD-mediated transcriptional arrest causes nucleolin to translocate from the nucleolus into the nucleoplasm within 2 h of treatment [52]. Indeed, after 2 h of ActD treatment, the tagged nucleolin protein showed re-localization to the nucleoplasm (Fig. 3d). The LIM and SH3 Protein 1 (LASP-1), tagged in the LASP-1-YFP-10×MS2 clone, is a member of the nebulin family of actin binding proteins. Treatment with Leptomycin B, a known inhibitor of nuclear export, interfered with the tagged protein export abilities, causing an increase in the nuclear signal of LASP-1-YFP (Fig. 3e).

### Using RNA FISH to detect endogenous transcription

The use of FISH probes to the MS2 region [54] is efficient and cost-effective for studies of several clones, since the probe targets all MS2-tagged alleles and therefore does not require unique synthesis of expensive probe sets per each gene sequence under study. However, a minimum number of MS2 repeats is required to yield detection of mRNA molecules. Clones with eight or less MS2 repeats showed negative staining in FISH using a 50-mer fluorescent probe to the repeated MS2 sequence. However, the tagged *LASP-1* gene with 10×MS2 repeats showed prominent active nuclear transcription sites, although single mRNA molecules were hard to detect (Supplementary Fig. S5). The *ANLN* gene in the ANLN-YFP-12×MS2 clone and the *IPO7* gene in the IPO7-YFP-20×MS2 clone both showed detectable transcription sites as well as single mRNAs by RNA FISH (Supplementary Fig. S5). Comparing the detection efficiency of the one MS2 probe (50-mer) that binds repeatedly to the MS2 repeats, to probe sets containing several short probes that target the whole MS2 region (20-mer, Stellaris) showed improved detectability for the latter (Supplementary Fig. S6a). As expected, no mRNA FISH signal was observed in cells that did not contain MS2-tagged genes (Supplementary Fig. S6b). Probe sets to the YFP region were also useful for all cell clones with eight and lower MS2 repeats (Supplementary Fig. S6c and d) and showed active transcription sites and single mRNAs.

**Figure 4:**
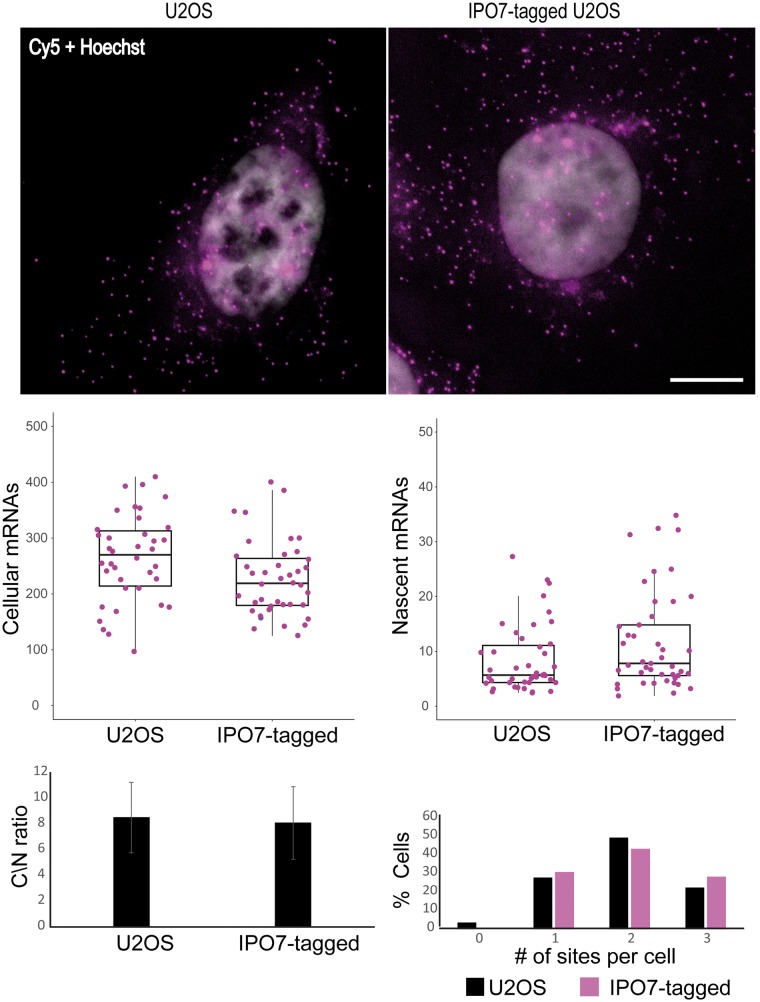
Comparative analysis between IPO7-tagged U2OS cells and untagged U2OS cells. **Top**, fluorescence microscopy images of the IPO7 mRNAs using Cy5-labeled-probes against the exons sequences in both tagged and untagged cells. Scale bar = 10 µm. **Bottom**, Plots displaying: cellular mRNA (tagged *n* = 40, untagged *n* = 38 cells, t-test *P* = 0.024), nascent mRNAs (tagged *n* = 87, untagged *n* = 74 active transcription sites, t-test *P* = 0.41311), cytoplasmic/nuclear ratio of mRNAs (tagged *n* = 40, untagged *n* = 38 cells, t-test *P* = 0.5044), and distribution of cells according to the number of active transcription sites (tagged *n* = 40, untagged *n* = 38 cells).

RNA FISH was used to examine whether the tag affects the transcriptional output of the targeted gene. We quantified the number of IPO7 transcripts in regular U2OS cells and in the IPO7-YFP-20×MS2 U2OS clone using probes against the IPO7 exon sequences. The values of cellular IPO7 mRNA numbers, nascent mRNAs, cytoplasm/nucleus mRNA ratios, and number of active transcription sites, were quantified, showing significant similarity between the tagged and untagged cells (Fig. 4). The total cellular numbers of IPO7 mRNA were statistically different between both cell types (*P* = 0.024); however, the difference was minor considering population heterogeneity.

Specificity of the tagged allele could be demonstrated by specifically knocking down the expression levels of one allele. shRNA plasmids targeting the YFP sequence showed efficient silencing of the YFP-tagged hnRNP A1, nucleolin, and *IPO7* genes, observed as a substantial decrease in the tagged protein levels (Supplementary Fig. S7).

### Detection and quantification of allele-specific expression

**Figure 5:**
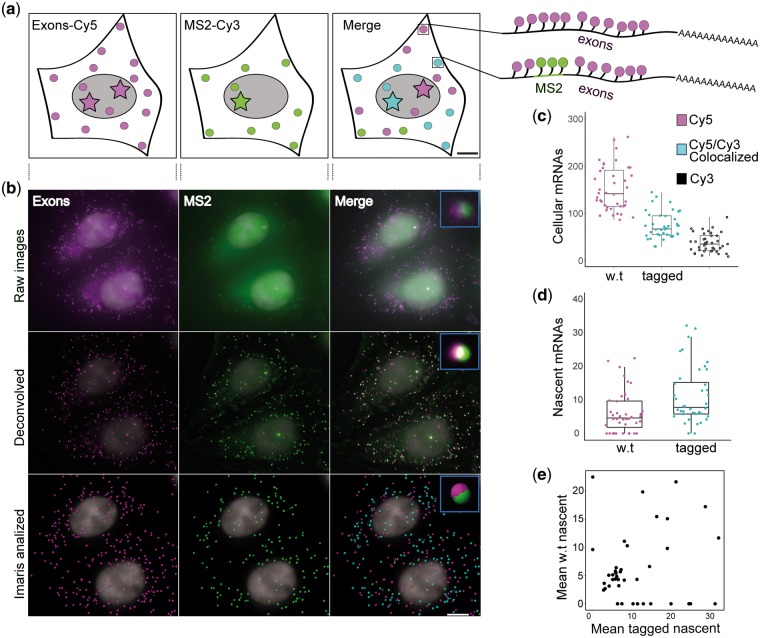
Allelic detection of the IPO7 gene and transcripts. (**a**) An illustration, showing CD-tagging-MS2’s ability to allow allelic detection using specialized fluorescent probes. Circle—single mRNP, star—active transcription at the transcription site, purple–Cy5, green—Cy3, cyan—co-localization of green and purple signals. (**b**) IPO7-YFP-20×MS2 cells were imaged using fluorescence microscopy. Images were deconvolved and taken for data analysis in Imaris software. The probes for the exons are labeled with Cy5 (purple) and the probes for the MS2 sequence are labeled with Cy3 (green.) Blue rectangles show enlarged co-localization events. Scale bar = 10 µm. (**c**) Cellular mRNA were quantified in IPO7-YFP-20×MS2 cells showing cellular mRNA average values (*n* = 40 cells). (**d**) Nascent mRNAs at the transcription site. There was no significant difference between the transcriptional activity of the alleles. t-test *P* = 0.0648 (*n* = 45 tagged, *n* = 43 w.t., active transcription sites). (**e**) Plot demonstrating the correlation between the tagged and w.t. alleles with regard to transcriptional activity. A correlation score of 0.74 was calculated. The calculation included only cells that had at least two active alleles (w.t. and tagged).

The CD-tagging-MS2 approach offers a unique opportunity to distinguish the transcriptional output of two endogenous alleles. Normally, it is difficult to visually distinguish between the alleles of the same gene since allele variability can occur on a single nucleotide level (e.g. SNPs). However, simultaneous use of fluorescent probes against the inserted tag (MS2) with probes targeting the gene exons, made it possible to identify and distinguish between the transcription sites of each allele and their transcribed mRNAs (Fig. 5a). Focusing on the IPO7-YFP-20×MS2 clone, IPO7 mRNAs were visualized using a combination of Cy5-labeled probes that complemented portions of exons 1 through 9 (labeled all alleles), and MS2 probes (Cy3-labeled), to detect the mRNAs transcribed from the tagged allele only (Fig. 5b). It should be noted that U2OS cells carry a triploid state of chromosome 11 on which the *IPO7* gene is located [61], therefore, some cells showed three active alleles of *IPO7*. We counted the number of mRNAs produced from each allele and quantified the number of nascent mRNAs on the transcription sites. The analysis showed that the number of cellular mRNAs produced from all three *IPO7* alleles was similar, thus each allele was responsible for approximately a third of the cellular IPO7 mRNA population (Fig. 5c and Supplementary Fig. S8a). This was confirmed RT-qPCR (Supplementary Fig. S8b). A small subpopulation of mRNAs detected only by the MS2 probe was also observed (see Discussion). The range of nascent mRNAs transcribed by tagged and untagged alleles was similar as well (Fig. 5d); this correlation was also seen when comparing the number of nascent mRNAs being transcribed on a tagged and untagged allele within the same cell (Fig. 5e).

**Figure 6:**
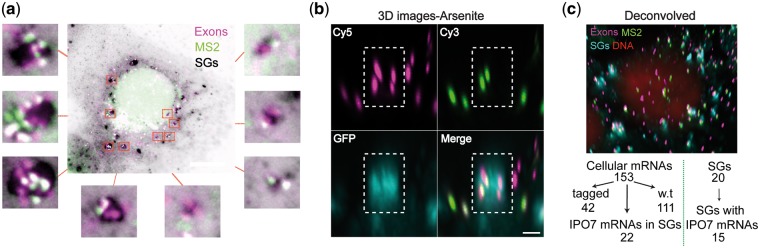
(**a**) IPO7-YFP-20×MS2 cells and the unique localization pattern of IPO7 mRNA observed under oxidative stress. To create a visible contrast between the mRNA and the SGs, the image of the SGs was inverted, presenting SGs in black. Scale bar = 10 μm. (**b**) 3D images that were constructed from 54 Z-stacks show the co-localization events from a side angle to verify that indeed the mRNAs resided within the SG. Scale bar = 1 µm. (**c**) An example for an analyzed cell that was treated with arsenite for 45 min. On the bottom, quantified data that was extracted from this cell. Scale bar = 10 μm.

**Figure 7:**
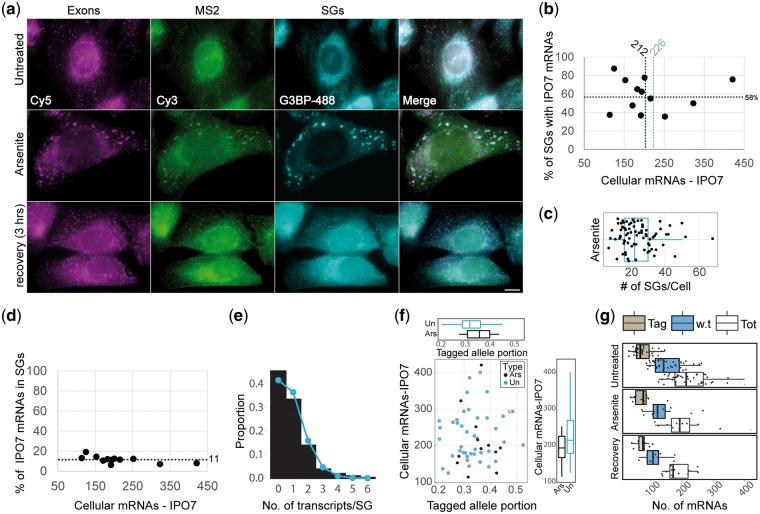
IPO7 mRNA distribution in SGs. (**a**) Cells from the IPO7 clone before and after a 45 min treatment with arsenite (1 mM, 45 min), and cells that were allowed to recover for 3 h. RNA FISH was performed with probes that differentiate between the tagged and w.t. alleles and was followed by immunofluorescence using the SG marker G3BP. Scale bar = 10 µm. (**b**) The percentage of SGs with IPO7 mRNA (*U* = 12 cells, 308 SGs). (**c**) The absolute number of SGs per cell (*n* = 80). (**d**) The portion of IPO7 mRNAs within SGs from the entire pool of IPO7 mRNAs (*n* = 12 cells). (**e**) Poisson distribution demonstrates random distribution into SGs under arsenite treatment (*P* = 0.337, *n* = 2545 IPO7 mRNAs, *n* = 308 SGs). The majority of SGs contain a single IPO7 mRNA. (**f**) Tagged allele portion from the entire pool of IPO7 mRNA. No notable change was observed. (**g**) IPO7-tagged U2OS cells underwent a 3 h recovery period from arsenite. Next, cellular mRNA was quantified with respect to allelic origin. The recovered cells maintained an allelic distribution which was similar to the untreated cells, throughout the stress response and recovery. The reduction in w.t. mRNAs following recovery had a *P* < 0.05.

Since the distribution of the tagged and untagged mRNAs seemed random in the cell, we wished to examine what is the fate of these mRNAs when the cells redistribute the cytoplasmic location of mRNAs, namely under stress conditions. It is suggested that SGs can harbor translationally stalled mRNAs for safekeeping until the stress passes [62], but the proportion of the mRNAs from the total population that actually enter SGs is not well characterized. We examined the distribution of the IPO7 mRNAs in the cytoplasm after exposure to arsenite, which causes oxidative stress, bringing to the formation of SGs. Single IPO7 transcripts were easily detected and quantified within SGs (Fig. 6a–c and Fig. 7a). The quantifications showed that some IPO7 mRNAs were distributed within 35–75% of the cell’s SGs with an average of 58% (Fig. 7b), which was equal to ∼14 SGs given that a typical cell contains ∼24 SGs under arsenite treatment (Fig. 7c). SG size increased over time as expected (Supplementary Fig. S9a), concurrently with a decrease in SG numbers per cell with time of arsenite treatment, supposedly due to fusion events [60]. Moreover, as SG size increased, the more IPO7 mRNAs appeared within (Supplementary Fig. S9b). The IPO7 cellular mRNA population remained unaffected by the stress conditions with an average of 212 mRNAs versus 226 in the untreated cells (Fig. 7b). Only a fraction, specifically 11% of the IPO7 mRNAs (Fig. 7d), were found in SGs upon arsenite treatment, which is equal to ∼23 mRNAs per cell. Examining the mRNA content of occupied SGs using Poisson distribution revealed a random distribution of mRNA within SGs (Fig. 7e).

To test for shifts in allelic mRNA balance during stress, we compared the tagged allele portion from the pool of IPO7 mRNAs in untreated cells and arsenite-treated cells. This analysis showed almost identical distribution of the tagged and untagged mRNAs for both conditions (Fig. 7f), implying that the mRNAs remaining in the cytoplasm and not confined to SGs, were not less protected than those mRNAs in SGs. Since the oxidative stress may not have an immediate effect on the RNA population, we examined whether there could be a delayed effect, which was tested by allowing the cells to recover from the stress for a period of 3 h, which eliminated all sign of SGs (Fig. 7a). The results indicated that IPO7 mRNA numbers were not affected during these treatments (Fig. 7g). Altogether, this analysis showed that the IPO7 mRNAs were randomly transcribed from all three alleles, that under stress conditions there was no preference in their distribution into SGs, and that the levels of mRNA remaining in the cytoplasm and not entering SGs, were not affected by the stress.

## Discussion

The CD-tagging-MS2 approach supports the identification of endogenous protein and mRNA distribution within the same cell, and can differentiate between the mRNA products of the different alleles of the same gene. The method exhibits physiological fidelity as was shown for several tagged genes, which retained known localization patterns on the protein level and proper splicing patterns on the mRNA level. The advantage of the CD-tagging-MS2 technique is that is ranks high in cost effectiveness, particularly when several mRNAs are being examined and compared. A similar approach has been performed previously in yeast cells, integrating an mCherry protein tag together with MS2 sequence repeats to tag endogenous genes [63]. Since yeast cells allow for easy genomic manipulation, this approach could successfully target specific genes, however, in mammalian cells this has been much more difficult. The CD-tagging-MS2 approach is relatively easy for mammalian cells, although tagging occurs randomly.

CD-tagging is cost effective and produces good quality signals for detecting differences in mRNA transcribed for different alleles. Pioneering studies have been able to distinguish between mRNA allelic origins using RNA FISH approaches. In one study, probes sets specific to the SNP sites of the *Nanog* gene were designed [45], while another study used short toehold probes complementary to regions in the mRNA which included single nucleotide variations (SNVs) in the *BRAF* gene and guide probes to help detect *bona fide* mRNAs [46]. Both methods require meticulous planning in order to design a correct set of probes for a specific nucleotide variation, whereas CD-tagging-MS2 simply requires the MS2 probe set and a standard set of probes complementary to the exons of the studied gene, since only a single allele is tagged with the MS2 region. Noteworthy, CRISPR technology can enable allele-specific tagging, as previously shown (64).

CD-tagging-MS2 efficiency is affected by multiple factors; virus packaging is influenced by plasmid purity, transfection effectiveness, packaging cells health, and Chloroquine concentrations, the latter a pH regulator that is added to the cells during transfection. Infection is influenced by virus lifetime, target-cell health and Polybrene concentrations, a positively charged polymer that facilitates viral entry. Integration events which lead to fluorescent tagging (0.1-1%) are influenced by multiplicity of infection (MOI), directionality of viral construct insertion relative to gene orientation, in-frame integration and gene expression/lack of expression in the target cells [65]. Moreover, 3′ and 5′ RACE are not sufficiently effective in identifying the tagged genes and therefore pose a limiting step. When a positive clone is obtained, it is important to validate that the tagged protein behaves similarly to the wild-type form. Other CD-tagging studies have generally shown that the inserted tag does not affect the protein, probably since protein domains tend to remain structurally independent to achieve structural stability that protects them from changes in the protein chain. Domains retain their function when isolated as fragments [66] and when combined with other domains from different proteins [67]. The path by which new protein domains are created may explain the cell’s “forgiveness” to an inserted tag domain. In accordance, our tests showed no deviation from current literature, and a previous study which used CD-tagging to create a library of more than 1200 clones labeled with YFP on the protein level, showed that the tagged proteins preserved their functionality [68–70]. Moreover, immunoblots of 20 different tagged proteins showed full-length fusions indicative to the non-protrusive nature of the method [37]. Nevertheless, protein structure, function, and localization might be altered due to the insertion of both the YFP and MS2 protein domains, and therefore the possible effects need to be examined for each case.

The loss of MS2 repeats during infection does not allow all clones to be used for MS2 FISH detection. Since, the reverse transcriptase (RT) of MLV has difficulty in polymerizing direct repeats during the transition from single strand RNA (ssRNA) to double strand DNA (dsDNA), which occurs in the cytoplasm of the infected cell prior to integration [71], we currently hypothesize that an integral RNase H activity of the RT enzyme is mainly responsible for the deletion events [72, 73]. The RT enzyme tends to fall from one template and switch to the other, which is identical sequence wise in the MLV virus. The switch is made by the already polymerized segment creating nucleotide complementation with the other template. However, when dealing with direct repeats such as the MS2 repeats, nucleotide complementation can occur on any repeat, which may result in skipped MS2 repeats aka repeat deletion. Future directions might include the use of the modified MS2 sequences [74] and RT mutated in its RNase H region together with wild-type RT, to reduce deletion frequencies while retaining infection efficiencies, as previously published [73].

The CD-tagging-MS2 system is unique in that it allows tagging endogenous genes on the mRNA and protein level simultaneously in mammalian cells. Moreover, CD-tagging-MS2 tags one allele only, creating a visually identifiable difference between tagged and untagged alleles that could be used for allelic discrimination by RNA FISH. Indeed, utilizing the difference between alleles by targeting probes against the exons of *IPO7* together with probes against the MS2 (or YFP) tag, we were able to examine the differences in activity of *IPO7* alleles. We found that the three alleles (including the tagged allele) independently produced similar levels of mRNA. Interestingly, a small population of MS2-tagged transcripts was also detected. These might be enhanced, in part, by deconvolution, however, we think they should not be addressed as false positive detection since they do not appear in the untagged U2OS cells. Hence, we suggest that these transcripts represent splice variants that bear the MS2 sequence (and therefore produce a prominent FISH signal) but lack enough exons to create a detectable exonic signal, or could be transcripts detected during the process of mRNA decay.

The single mRNA detection allowed us to count endogenous mRNAs inside SGs [60]. In early studies, poly(A)+ RNA or β-globin mRNA[75, 76] were identified inside SGs, thus visualizing the SG-RNA relationship proposed for these structures [77]. However, the RNA was typically identified as clusters within SGs without single molecule resolution. One study measured the overall intensity of MCP-GFP clusters by tagging β-Gal-MS2 mRNA in the cytoplasm of HeLa cells under arsenite conditions. To evaluate what percentage of β-Gal mRNAs was entering SGs, the signal from the RNA clusters was compared to the signal from the cytoplasm. They showed that only 7.3% of total β-Gal-mRNA entered SGs [78]. In another study, single molecules of β-actin mRNAs within SGs were monitored and quantified using multiply-labeled tetravalent RNA imaging probes (MTRIPs) [79]. This study showed that only 3.7% of β-actin mRNAs were localized in SGs during arsenite treatment. The results we obtained for the IPO7 mRNAs are in scale with these measurements. After allowing the cells to recover, we were able to examine whether oxidative stress alters the allelic balance of mRNAs in cells. The results suggest otherwise and raise interesting questions regarding the stress response, for instance; why is only a small portion of each mRNA population studied thus far targeted to SGs?; Why is this portion of IPO7 SG-targeted mRNAs constant between cells that actually differ significantly in their total number of IPO7 mRNA numbers? Altogether, this simple and cost-effective endogenous tagging approach allows the detection of endogenous protein and mRNA, on an allelic level, in single cells.

## Supplementary Material

Supplementary Data
