# Supplementary material for: CD-tagging-MS2: detecting allelic expression of endogenous mRNAs and their protein products in single cells
Source: Biol Methods Protoc. 2017 May 12;2(1):bpx004. doi: 10.1093/biomethods/bpx004 (PMC6994078; doi:10.1093/biomethods/bpx004)
Supplement: Supplementary Data [file bpx004_supp.zip › bpx004-suppl_data/Supplementary figures Sheinberger et al.DOCX]

**SUPPLEMENTARY INFORMATION**

**CD-tagging-MS2: detecting allelic expression of endogenous mRNAs and their protein products in single cells**

Jonathan Sheinberger, Hodaya Hochberg, Erez Lavi, Itamar Kanter, Shira Avivi, Gita Reinitz, Avital Schwed, Yuval Aizler, Eli Varon, Noa Kinor, and Yaron Shav-Tal


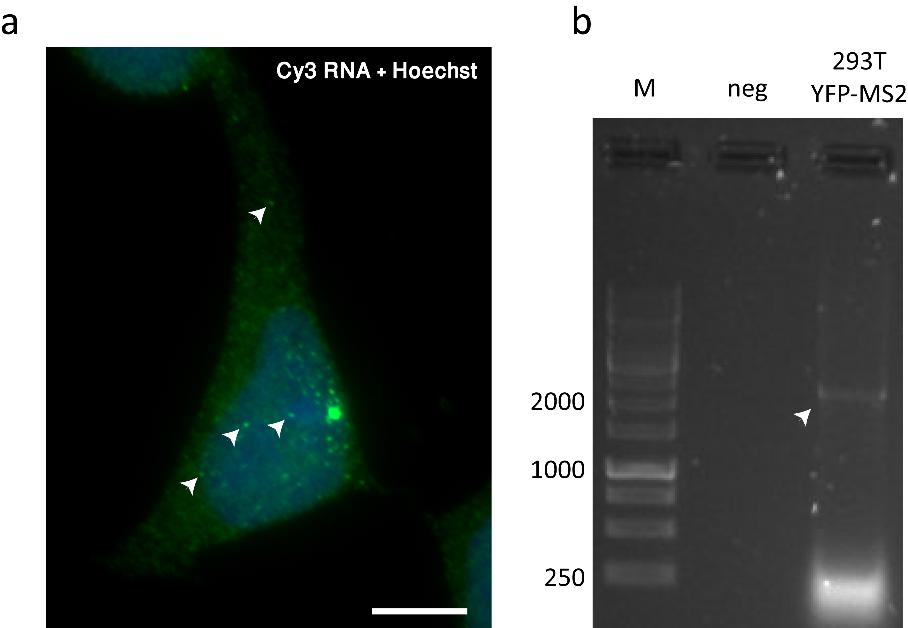


Supplementary Figure S1. HEK 293T cells stably expressing the CD-tagging-MS2 cassette. (**a**) RNA FISH using with probes that bind to the MS2 repeats (50 mer, green). White arrowheads point to single transcripts. Scale Bar= 10 µm. (**b**) PCR using primers that flank the YFP-24xMS2 region was performed on DNA extracted from the HEK 293T cells. The expected length is 2293 bp when all 24 repeats are present.


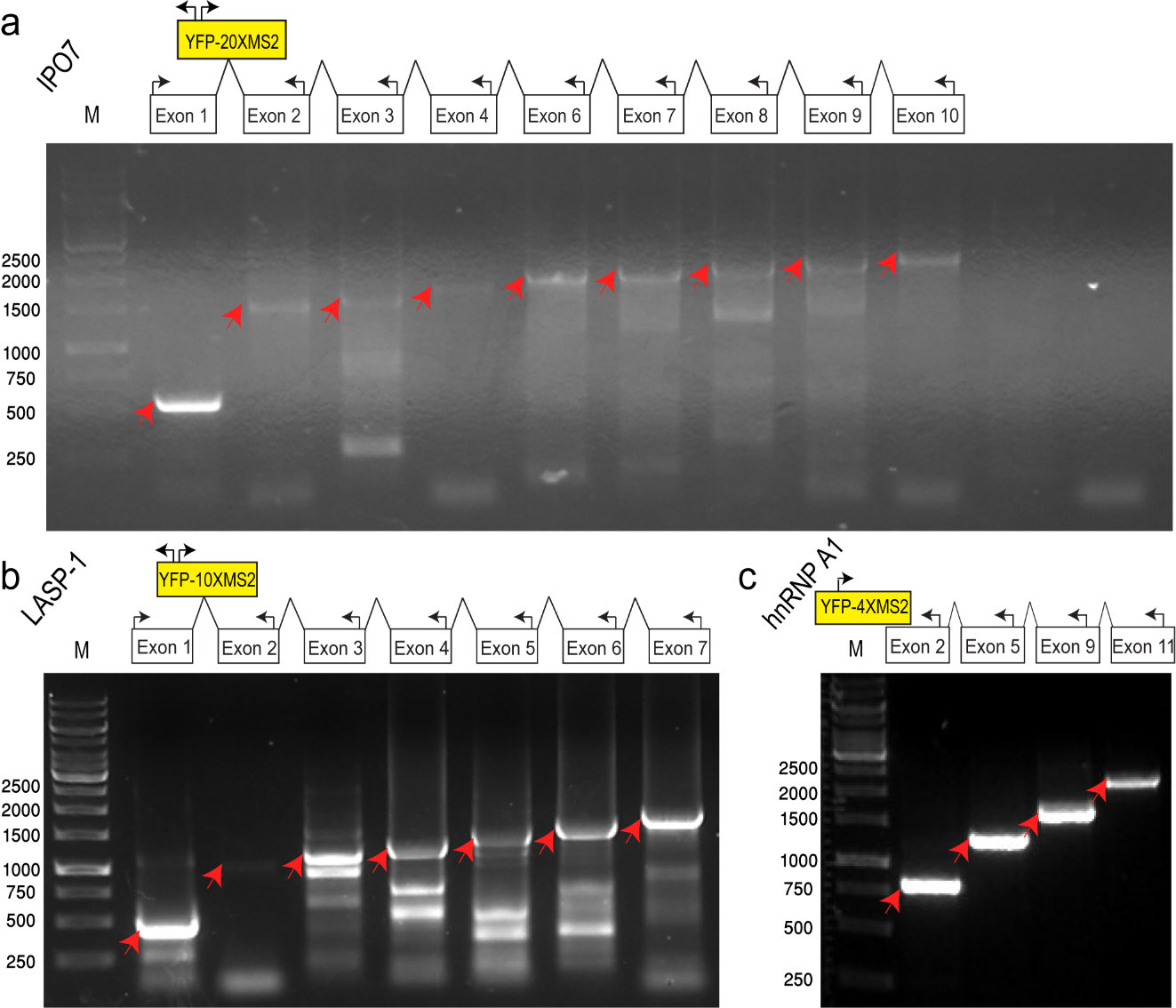


Supplementary Figure S2. Examining the splicing pattern of the tagged genes. RT-PCR reactions with primers for walking along the mRNA. (**a**) IPO7-YFP-20xMS2, (**b**) LASP-1-YFP-10xMS2, (**c**) hnRNP A1-YFP-4xMS2. The bands that represent the predicted size of the mRNA including the insertion are marked with red arrows.


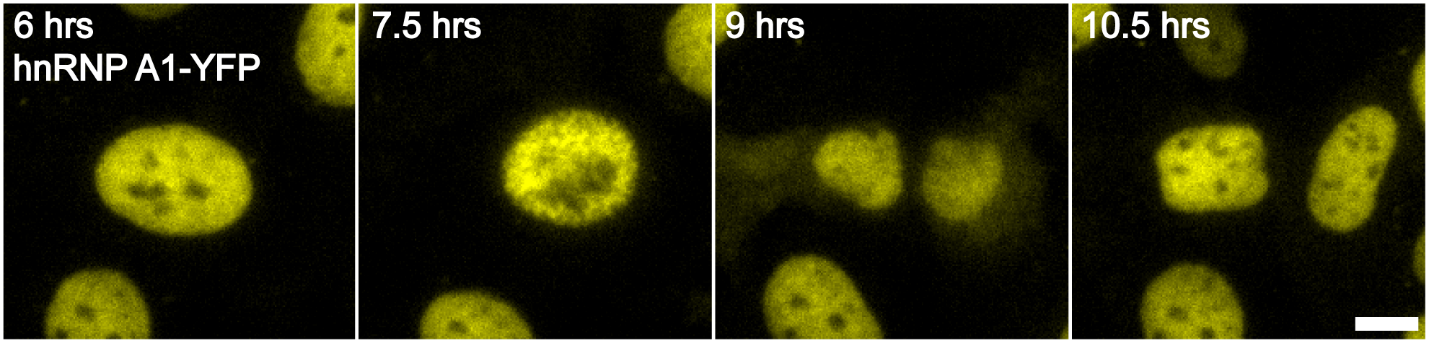


**Supplementary Figure S3.** Frames from a time-lapse movie of hnRNP A1-YFP-4xMS2 cells during mitosis. Images were captured every 30 minutes using the YFP channel. Scale bar = 10 µm.


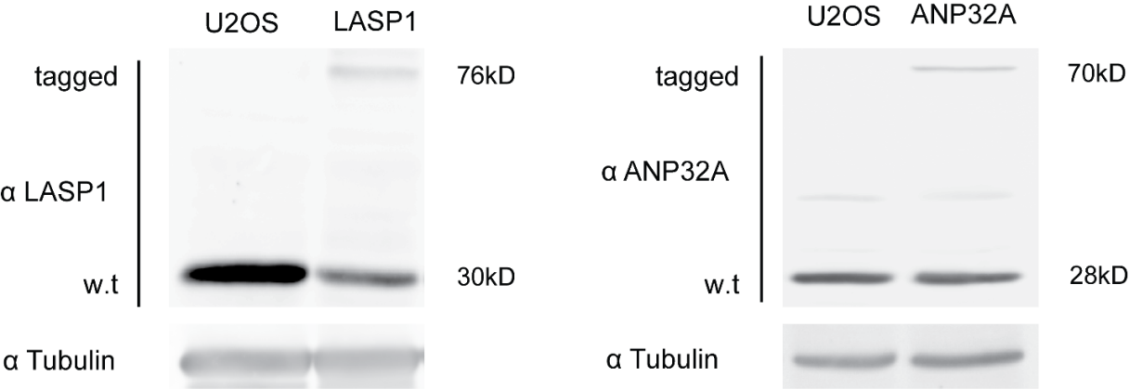


**Supplementary Figure S4.** Protein extracts from the LASP-1-10xMS2 and ANP32A-8xMS2 clones underwent western blotting to detect the tagged and wildtype proteins. Western blotting was performed using antibodies that recognized both the tagged and wildtype proteins.


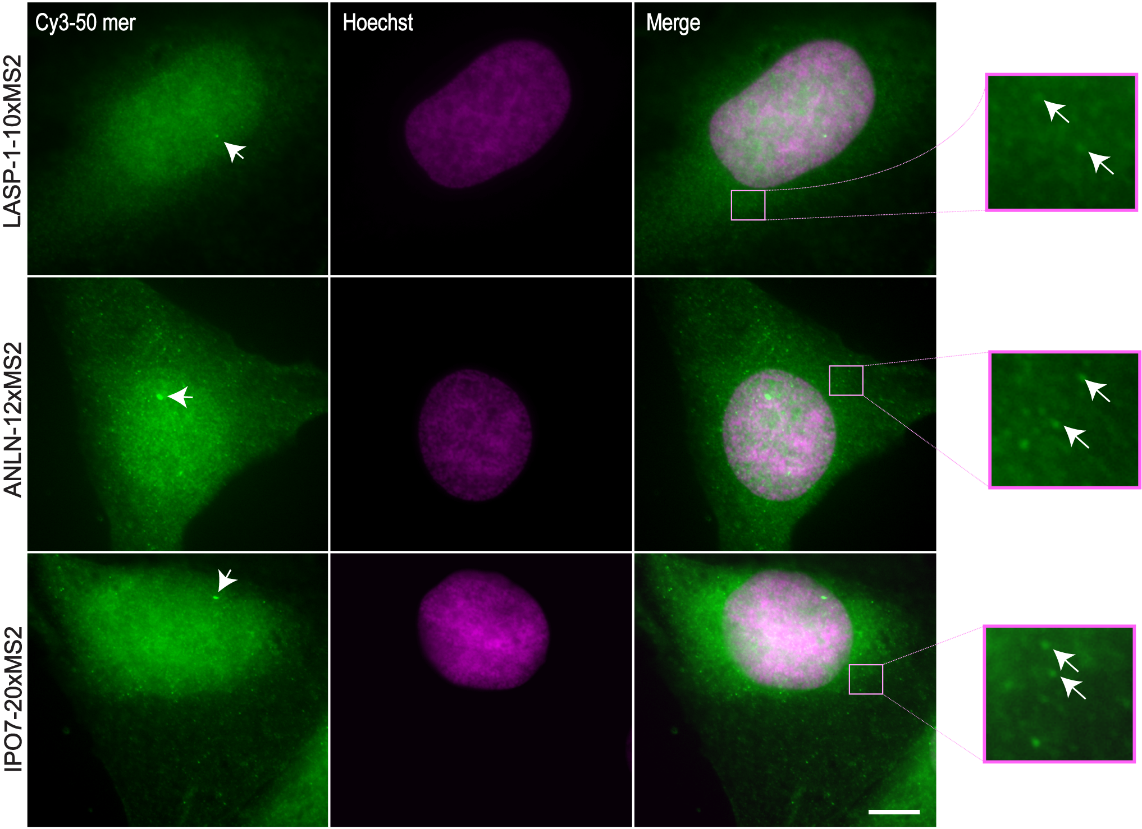


**Supplementary Figure S5.** Detection of MS2-tagged mRNAs on active genes and throughout the cells. RNA FISH images of LASP-1-10xMS2, ANLN-12xMS2 and IPO7-20xMS2 cells. Arrows point to active transcription sites. Arrows in enlarged areas point to single mRNAs. Scale bar= 10 µm.


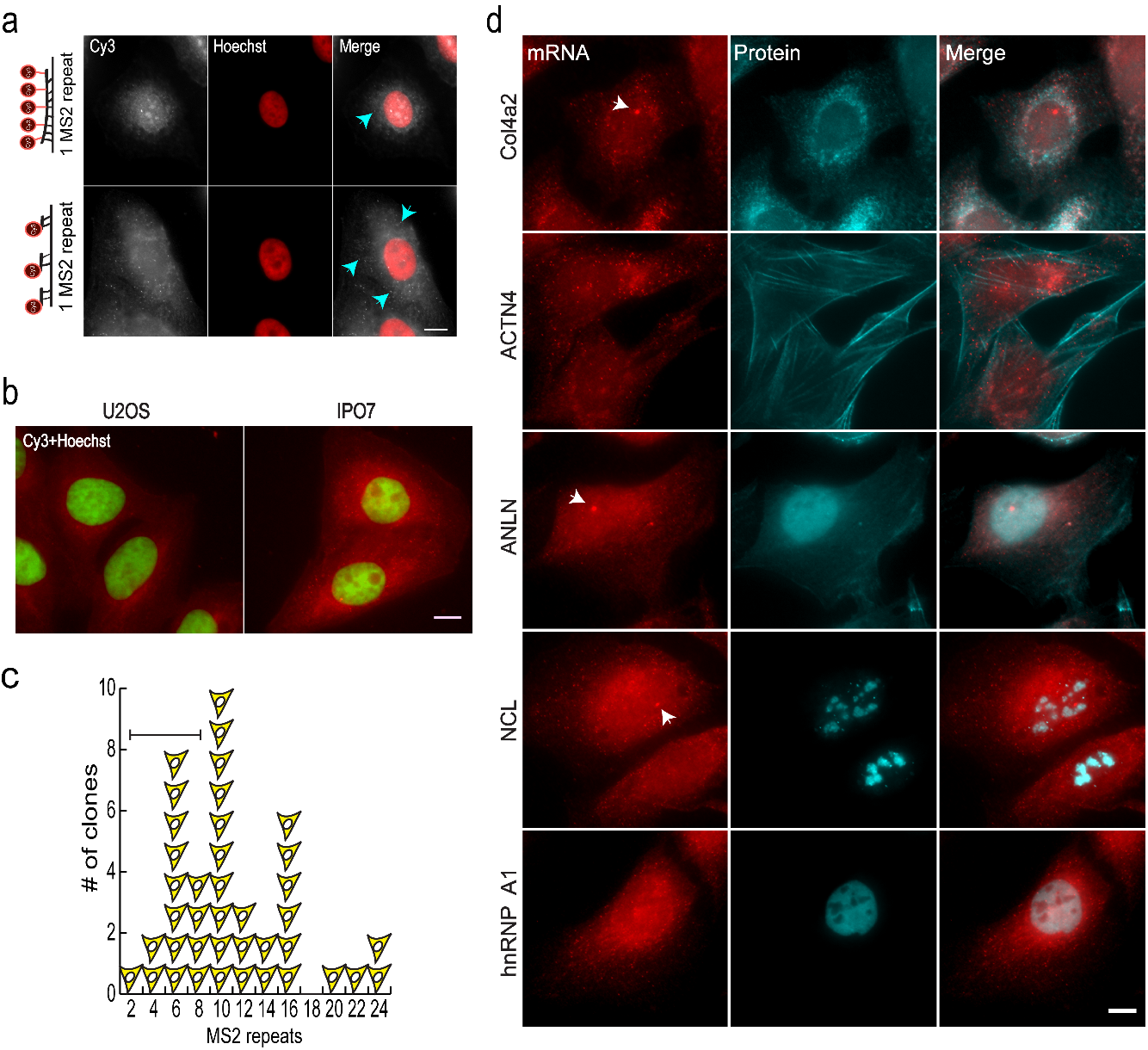


**Supplementary Figure S6.** Detection of single mRNAs by RNA smFISH. (**a**) RNA FISH with one MS2 probe (top, 50 mer) compared to three MS2 probe sets (bottom, 20 mers, Stellaris) on clone IPO7-YFP-20xMS2 using the Stellaris FISH protocol. Cyan arrowheads point to single mRNAs. (**b**) RNA FISH using 20 mer MS2 probe sets in U2OS and in IPO7-tagged U2OS cells. (**c**) Chart representing the number of MS2 repeats integrated into the different genes, from DNA-based PCR (n=40). (**d**) RNA FISH probe set to the YFP sequence (red) enables the detection of actively transcribing genes even in clones that possess low MS2 repeat numbers: Col4a2-2xMS2, ACTN4-4xMS2, ANLN-12xMS2, NCL-4xMS2 and hnRNP-A1-4xMS2. The fluorescence of the endogenously YFP-tagged protein (pseudo-colored cyan) is preserved. Arrows point to active transcription sites. Scale bar= 10 µm.


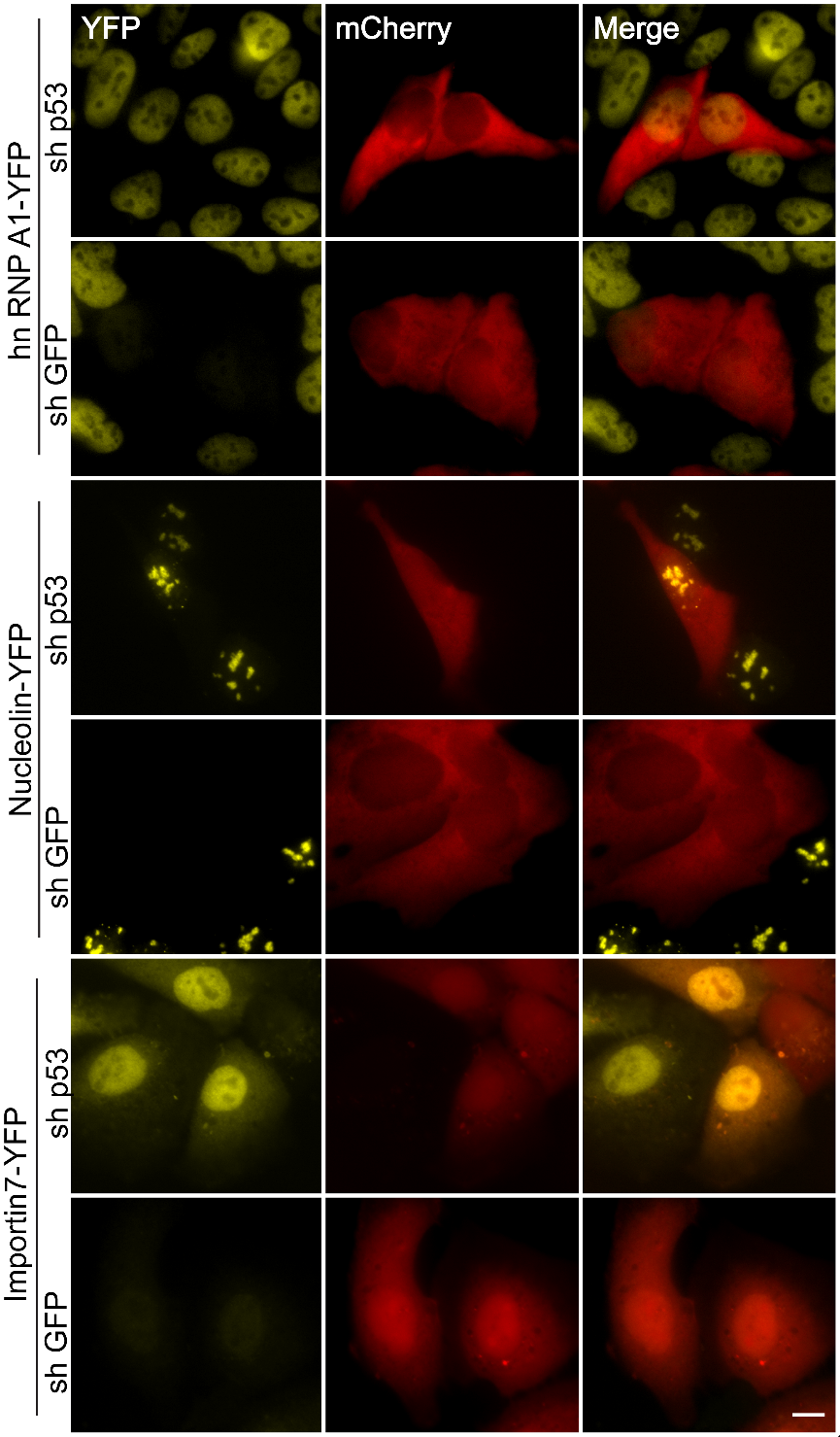


**Supplementary Figure S7.** shRNA experiments performed on three tagged clones showing high efficiency of tagged mRNA knockdown. hnRNPA1-4xMS2, nucleolin-4xMS2 and IPO7-20xMS2 tagged clones underwent co-transfection with a plasmid that transcribes shRNA to YFP and with an mCherry gene (hnRNPA1 and nucleolin) or a Cerulean-plasmid gene (IPO7, pseudo colored red) serving as an indicator for cells expressing the shRNA sequences (red), as well as the levels of shRNA expression. A similar plasmid expressing shRNA sequences against the p53 gene served as a control. Scale bar= 10 μm.


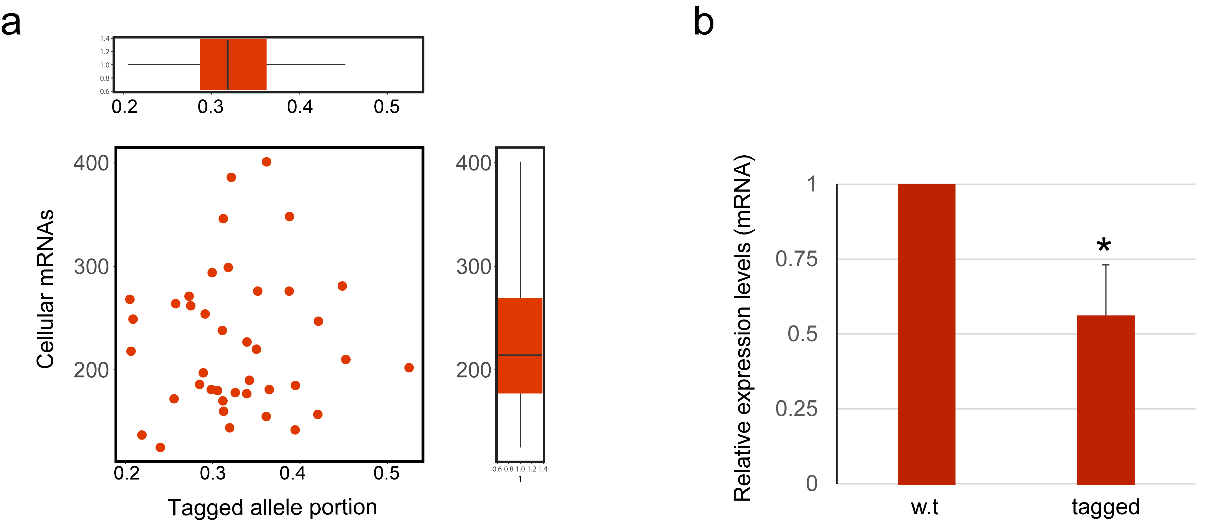


**Supplementary Figure S8.** (**a**) The relative portion of cellular mRNAs transcribed from the tagged allele from the entire IPO7 mRNA pool (n=40 cells). A correlation score of 0.047 was calculated. (**b**) RT-qPCR analysis of tagged and wildtype mRNA for the *IPO7* gene. Specific primers for each transcript (tagged and w.t) were used to quantify the ratio between the transcripts. *P*= 0.011 (n=3).

**
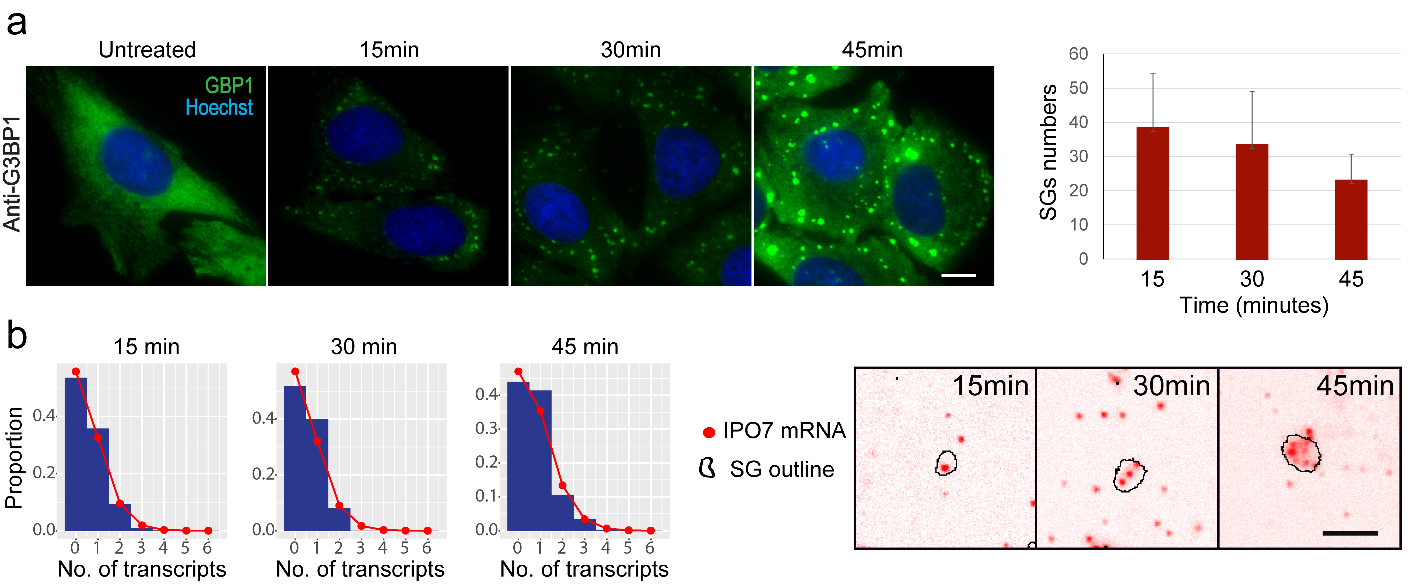
**

**Supplementary Figure S9.** SGs and mRNA behavior during 45 minutes of cellular stress. (**a**) U2OS cells were treated with arsenite for the indicated times. Then, immunofluorescence was performed using the anti-G3BP1 marker for stress granules. SGs were counted using ImageJ. A significant reduction was registered for the transition from 30 to 45-treatment time. *P*=0.0055 (n=26). Scale bar= 10 µm. (**b**) Left, Poisson distribution showing an increase in SGs that harbor more than 1 IPO7 mRNA over time of arsenite treatment. Right, representative example of the mRNA capacity of an SG, over time of arsenite treatment and SG size. Scale bar= 3 µm.

**
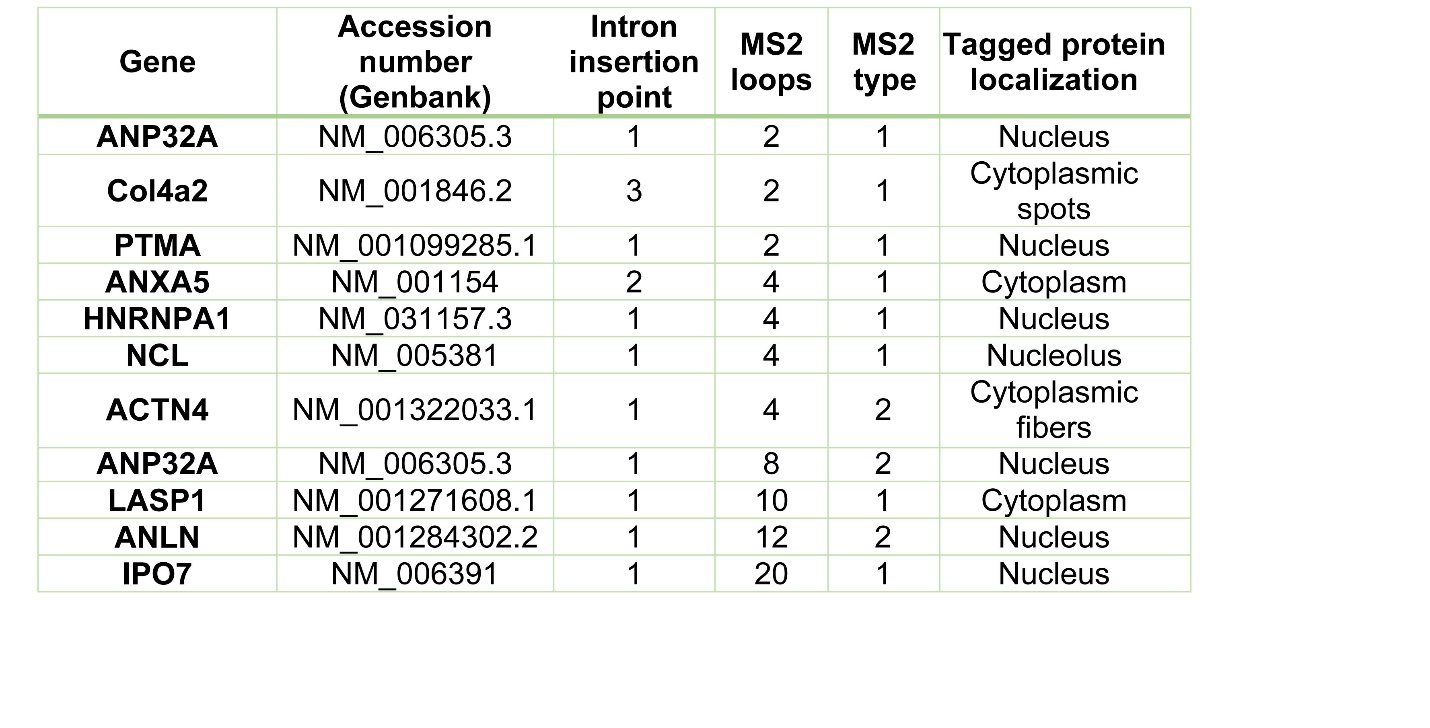
**

**TABLE S1**. List of identified clones showing the gene name, the location of intron into which inserted, the number of MS2 repeats in the genome, and the MS2 gene construct used (1^st^ or 2^nd^ generation).

**Supplementary video legends**

**Supplementary video S1**. A montage of six library clones showing normal cell divisions. For all six clones, an image of the endogenous protein was captured every 30 minutes for 720 min.

**Supplementary video S2.** Time-lapse imaging showing the response of hnRNP A1-YFP cells (from figure 3b) to 1mM arsenite treatment and the formation of stress granules. Images were acquired every 5 minutes for 105 min.

**Supplementary video S3.** Time-lapse imaging showing the response of Nucleolin-YFP cells to ActD treatment (5 μg/ml) and the disassembly of nucleoli. Images were acquired every 10 minutes for 490 min.
